# Clinical characteristics and outcomes of non-tuberculous mycobacterial pulmonary infections after hematopoietic stem cell transplantation: A retrospective cohort study

**DOI:** 10.1016/j.htct.2025.103841

**Published:** 2025-05-09

**Authors:** Zahia Esber, Hamza Salam, Shefali Godara, Ayman Soubani

**Affiliations:** Internal Medicine, Division of Pulmonary, Critical Care and Sleep Medicine, Wayne State University School of Medicine, Detroit, MI, USA

**Keywords:** Non-tuberculous mycobacteria pulmonary infection, Non-tuberculous mycobacteria lung disease, Hematopoietic stem cell transplantation

## Abstract

**Introduction:**

Non-tuberculous mycobacterial infections are rising as complications of bone marrow transplantation with lung disease being the most common clinical presentation. The identification and management of these infections in hematopoietic stem cell transplantation patients remains underrecognized. This study aims to investigate the clinical characteristics and outcomes in patients with post-transplant pulmonary infections.

**Methods:**

The charts of 3,000 adult patients who received transplants over 11 years at the Karmanos Cancer Institute, a tertiary-care cancer center in Detroit, were reviewed. The diagnoses of post-transplant pulmonary non-tuberculous mycobacterial infections of 51 patients were defined as definite, probable or possible based on the American Thoracic Society (ATS) and Centers for Disease Control and Prevention guidelines. The identified organisms were further characterized as rapid- or slow-growing mycobacteria. Clinical characteristics, risk factors, microbiologic data, therapy and outcomes of the patients were collected and analyzed.

**Results:**

About half (*n* = 26; 51%) of the patients were identified with definite pulmonary infection. There was a trend of cardiovascular and pulmonary comorbidities in these patients. The majority (*n* = 44; 86.3%) were on steroid and immunosuppressive therapy in the setting of graft-versus-host disease. The most common presenting symptoms were a combination of change in cough and worsening shortness of breath. The most common radiologic pattern was nodular infiltrates in 15 (29.4%) patients. *Mycobacterium avium complex* was identified in 38 (74.5%) patients. The majority of patients with these infections (76.5%) did not receive antimycobacterial therapy. Survival was reported in 42 (82.4%) patients.

**Conclusion:**

Outcomes vary significantly among non-tuberculous mycobacterial pulmonary infections based on mycobacterial species, rate of colonization and degree of immunosuppression. The prognosis is overall good due to slow growing mycobacteria. Prospective multicenter studies are required to further guide the management of these patients.

## Introduction

Non-tuberculous mycobacterial (NTM) infections are surging as complications of organ transplantation with lung disease being the most common clinical presentation. The identification and management of these infections in patients after hematopoietic stem cell transplantation (HSCT) is underrecognized. This study aims to investigate the clinical characteristics and outcomes of patients with these infections.

## Patients and methods

Karmanos Cancer Institute (KCI) is a tertiary-care cancer center in Detroit. Medical records were reviewed to identify all over 18-year-old patients who underwent HSCT at KCI over an 11-year period. Data collection including age, gender, cardiovascular and pulmonary comorbidities, symptoms, radiologic patterns, microbiologic data, immunosuppressive therapy and outcomes.

Microbiology laboratory records were examined to identify patients with positive mycobacterial cultures obtained from sputum, bronchoalveolar lavage, or other clinical specimens throughout the 11-year study period. NTM infections were defined by the American Thoracic Society (ATS) and Centers for Disease Control and Prevention guidelines. Mycobacteria specimens had been cultured, stained and identified by conventional growth in special media and biochemical conditions. All computed tomography scans (CT) of the thorax were reviewed for abnormal radiologic patterns, with findings being characterized as lung nodules, consolidation, bronchiectasis, interstitial pattern, pleural effusion and mediastinal lymphadenopathy. This cohort comprised patients who fulfilled ATS criteria for NTM lung disease, including: (1) chest radiograph or, in the absence of cavitation, chest high-resolution computed tomography (HRCT) scan; (2) three or more sputum specimens for acid-fast bacilli analysis; and (3) exclusion of other disorders. The following criteria applied to symptomatic patients: radiographic opacities (nodular or cavitary) or an HRCT scan that shows multifocal bronchiectasis with multiple small nodules. These criteria fit best with *Mycobacterium avium complex* (MAC), *M. kansasii*, and *M. abscessus*. Not enough is known about most other NTM to be certain that these diagnostic criteria are universally applicable to all NTM respiratory pathogens [1] The NTM organisms were further characterized by their pathogenicity and identified as rapid- growing mycobacteria (RGM) and slow-growing mycobacteria (SGM) [2].

## Results

This retrospective study comprised 51 (1.7%) of 3000 adult patients who were diagnosed with NTM infections after HSCT over the 11-year period. All 51 patients met one or more of the ATS clinical, radiologic or microbiologic criteria for NTM lung disease. Thirty-two (62.7%) of the 51 patients were male with a median age of 51 years (range: 20–77). Ten (19.6%) and five (9.8%) had cardiovascular and pulmonary comorbidities, respectively. MAC was identified in 38 (74.5%) patients, *M. gordonae* in seven (13.7%), *M. chelonae* in two (3.9%), and other mycobacteria in four (7.8%) patients. Twenty-six (51%) had definite diagnoses of NTM pulmonary infections with clinical, radiological and culture evidence. Eighteen patients (35.3%) had possible NTM infections with only positive cultures, and one (2%) had probable NTM infection due to radiological and culture evidence without any clinical manifestations. Mycobacterial cultures were positive in 23 (45.1%) bronchoalveolar lavage (BAL) cultures and in 22 (43.1%) sputum cultures (Table 1).

**Table 1:** Clinical characteristics of the patients.

Variable	*n* = 51
Sex - n (%)	
Male	32 (62.7)
Female	19 (37.3)
Age, years – median (range)	51 (20–77)
Clinical manifestations - *n* (%)	32 (62.7)
Worsening shortness of breath	7 (13.7)
Cough and worsening shortness of breath	10 (19.6)
Fever	3 (5.9)
Weight loss	1 (2.0)
Hemoptysis with worsening shortness of breath or weight loss or fever	3 (5.9)
Abnormal radiologic pattern on CT thorax - *n* (%)	
Lung nodules	15 (29.4)
Consolidation	7 (13.7)
Bronchiectasis	4 (7.8)
Interstitial pattern	4 (7.8)
Lung nodule, mediastinal lymphadenopathy and pleural effusion	1 (2.0)
Mycobacterial identification - *n* (%)	
*Mycobacterium avium complex*	38 (74.5)
*Mycobacterium gordonae*	7 (13.7)
*Mycobacterium chelonae*	2 (3.9)
*Other mycobacteria*	4 (7.8)
Steroid and immunosuppressive therapy - *n* (%)	44 (86.3)
Antimycobacterial therapy for definite NTM infection - *n* (%)	
Complete therapy (*n* = 26)	6 (23.1)
Incomplete therapy (*n* = 26)	20 (76.9)

Clinical manifestations were reported in 32 (62.7%) of the 51 patients. Of these, ten (19.6%) had both changes in cough and worsening shortness of breath, seven (13.7%) had worsening shortness of breath only, three (5.9%) had fever, one (2%) had weight loss and three (5.9%) had hemoptysis with worsening shortness of breath or weight loss or fever. The most common abnormal radiologic finding on thorax CT was lung nodules in 15 (29.4%) patients; seven (13.7%) had consolidation, four (7.8%) had bronchiectasis and four (7.8%) had an interstitial pattern. Only one patient (2%) had lung nodules, mediastinal lymphadenopathy and pleural effusion. Twenty patients (39.2%) had normal thorax CT results.

**Figure 1:**
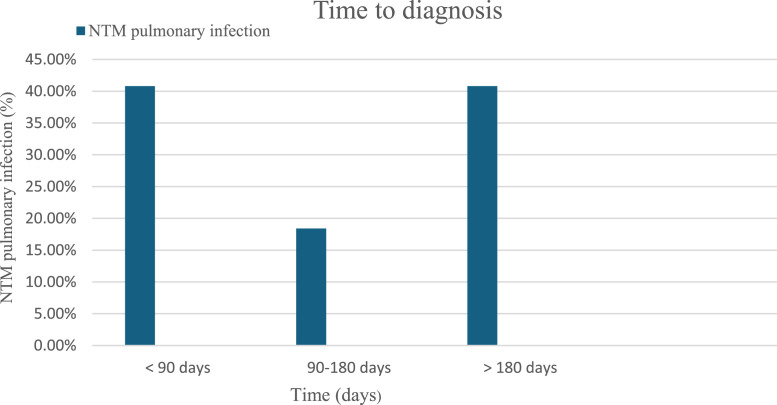
Time to diagnosis.

The median time until diagnosis of NTM pulmonary infections after the HSCT was greater than 180 days in 20 patients (40.8%), and within 90 days in 20 (40.8%) patients. Only nine patients (18.4%) were diagnosed with NTM pulmonary infection between 90 and 180 days after HSCT (Figure 1). The majority, 44 patients (86.3%), were on steroid and immunosuppressive therapy in the setting of graft-versus-host disease (GvHD). Only six (11.8%) completed antimycobacterial therapy, whereas six (11.8%) had incomplete therapy and 39 (76.5%) did not receive any antimycobacterial therapy. Survival was reported in 42 (82.4%) patients with post-HSCT NTM pulmonary infections.

## Discussion

This study found that the majority of the patients who were diagnosed with NTM after HSCT were male, had a median age of 51 years, and had pulmonary and cardiovascular comorbidities. In a cohort study by Liu et al., patients aged >45 years with extensive GvHD were at increased risk of mycobacterial infections [3]. Pre-transplant T cell depleting therapy increases the risk of mycobacterial infections [4]. Epidemiologic studies of survivors have shown that HSCT is associated with higher cardiovascular risk partially due to exposure to cardiotoxic chemotherapy and radiation, as well as detrimental direct and indirect effects on the cardiovascular reserve [5]. In the present cohort, the majority (*n* = 44; 86.3%) were on steroid and immunosuppressive therapy in the setting of GvHD. We believe that the increased incidence of cardiovascular disease in this population is related directly or indirectly to chemotherapy and radiation and its deleterious effects on the immune system in HSCT patients.

In two reviews in 1994 and 1997, NTM infections were rare, occurring in only 0.37% and 0.40% of adult patients who received HSCT, respectively [6,7]. A recent report from other centers indicate that in 2003, the rate of NTM infection was 5 to 20 times higher in a cohort of 50 patients following allogeneic HSCT [4]. In the present cohort, NTM infection occurred in 1.7% of adult HSCT patients. The higher rates of NTM infection may have resulted from an increased recognition and improved identification in mycobacterial cultures [1]. Therefore, NTM infection after HSCT must be recognized as a complication that requires heightened awareness to identify risk factors with timely diagnosis and therapy.

Half (51%) of the patients in the current study had definite pulmonary mycobacterial infections. Of the 25 HSCT patients who had probable or possible NTM infections, 18 (35%) had only positive cultures which solely cannot differentiate between NTM colonization and infection, but can affect the decision to initiate antimycobacterial therapy. Another important factor for treating NTM infections is the identification of NTM species. Most of the identified species in this study were slow-growing mycobacteria. *M. gordonae*, the most frequently isolated mycobacterial, was found in seven (13.7%) patients.

The time to diagnosis after the transplant was either within 90 days (40.8%) or after 180 days (40.8%) in this current study. In a large 2000 retrospective study spanning 20 years, the median time to diagnosis of lower respiratory tract NTM disease following HSCT was 251 days for SGM and 61 days for RGM [6]. The infections could occur early in the pre-engraftment phase of HSCT, or later while receiving immunosuppressive therapy for GvHD [8,9].

The clinical characteristics of pulmonary NTM infections differ between patients with acquired immunodeficiency syndrome (AIDS), and immunocompromised hosts after transplantation and, evidently, these infections different from immunocompetent patients. In the present population, the most common single clinical manifestation of NTM infection after HSCT was deteriorating shortness of breath occurring in seven (13.7%) of the patients, while only three (5.9%) had fever. The combination of change in cough and worsening shortness of breath was the most common presenting manifestation in this population in ten (19.6%) of 32 patients with three (5.9%) patients having hemoptysis with worsening shortness of breath, weight loss or fever. These clinical characteristics suggest that the majority of patients who lack systemic symptoms of hemoptysis and weight loss do not require antimycobacterial therapy. Clinical suspicion based on symptoms alone is low because NTM infection after HSCT is difficult to diagnose due to lack of specific symptoms and atypical presentation [10].

Studies with high resolution CT of the chest have shown that up to 90% of patients with mid- and lower-lung field noncavitary disease with MAC also have multifocal bronchiectasis [1]. There was no specific radiologic finding in the current cohort although the most common radiologic pattern identified in HSCT patients with NTM infections was lung nodules in 15 (29.4%) of the 51 patients. Seven (13.7%) patients had consolidation and four (7.8%) patients each had bronchiectasis and interstitial pattern. Twenty (39.2%) of the 51 patients in this current study had normal thorax CT.

The most commonly NTM isolated in this population was MAC in 74.5% followed by *M. chelonae* in two (3.9%) patients, and *M. gordonae* in seven (13.7%). These mycobacterial species are all SGM; *M. gordonae* is non-pathogenic and is known as a weak mycobacterium that rarely causes overt disease or requires therapy [1]. However, no RGM, such as *M. abscessus*, which are intrinsically resistant and difficult to treat, were identified in this current study.

Initiation of antimycobacterial therapy is a concern because of multiple factors including 1) adverse effects of antimycobacterial therapy, 2) drug-drug interactions with drug prophylaxis after HSCT, and 3) prolonged duration of therapy until culture conversion [1]. In the present cohort, only six of 26 patients (23.1%) with definite NTM infections completed antimycobacterial therapy, whereas 20 (76.9%) did not complete antimycobacterial therapy. The prognosis of pulmonary NTM infections in transplant patients is overall favorable. Most patients (82.4%) with definite, probable or possible NTM infections in this study are alive and 25 of 51 (49.1%) did not receive any antimycobacterial therapy. A high survival rate is noted in patients with NTM infections after HSCT while only 23.1% completed antimycobacterial therapy. This is related to (1) the nature of the disease due to predominantly SGM MAC which is a less aggressive disease than RGM, (2) the paucity of patients with systemic symptoms (only 5.9% had fever and hemoptysis with worsening shortness of breath or weight loss or fever) and (3) the predominant radiologic patterns of nodularity and bronchiectasis which correlates with less severe disease than cavitary lesions [1].

Our study had several limitations. First, this is a single-center retrospective and uncontrolled study which could affect the validity of the results. Secondly, the study included non-pathogenic SGM which could contribute to a favorable outcome. Third, four of the NTM culture results were not identified as to the specific species. Different species have different clinical impacts with the SGM causing less aggressive mycobacterial disease than the RGM. Lastly, data about potential use of one or more antibiotics of the antimycobacterial therapy such as macrolide agents is lacking in this study. However, it is unlikely that any of our patients received any effective triple antimycobacterial therapy empirically without having a definite NTM infection, and single macrolide therapies are not effective in NTM infections.

## Conclusion

Clinical characteristics of NTM pulmonary infections after HSCT are nonspecific and require a high index of suspicion to make the diagnosis of definite NTM pulmonary infections. Overall NTM infections due to SGM in post-HSCT patients have a favorable prognosis. Prospective multicenter studies are required to guide further management of different NTM infections after HSCT.

## Funding

This research did not receive any specific grant from funding agencies in the public, commercial, or not-for-profit sectors.

## Authors contribution

(1) The conception and design of the study: AS
(2) Acquisition of data: Dr Salam, SG
(3) Analysis and interpretation of data, drafting the article or revising it critically for important intellectual content: ZE, AS
(4) Final approval of the version to be submitted: AS, ZE

## Conflicts of interest

The authors declare no conflicts of interest.

## References

[1] Griffith D.E., Aksamit T., Brown-Elliott B.A., Catanzaro A., Daley C.L., Gordin F. (2007). An official ATS/IDSA statement: diagnosis, treatment, and prevention of nontuberculous mycobacterial diseases. Am J Respir Crit Care Med.

[2] Rocha H.A.L., Almeida M.E., Silva A.R., Rodrigues F.J., Costa L.M., Pereira M.S. (2018). Effectiveness of rapid response teams in reducing intrahospital cardiac arrests and deaths: a systematic review and meta-analysis. Rev Bras Ter Intensiva.

[3] Liu Y.C., Hung C.C., Chen C.Y., Huang C.T., Lin C.F., Chou C.H. (2020). Mycobacterial infections in adult recipients of allogeneic hematopoietic stem cell transplantation: a cohort study in a high endemic area. J Microbiol Immunol Infect.

[4] Weinstock D.M., Brown A.E., Petropoulos C.J., Wagner B., Mena-Romo M.A., Sepkowitz K.A. (2003). High rates of infection and colonization by nontuberculous mycobacteria after allogeneic hematopoietic stem cell transplantation. Bone Marrow Transplant.

[5] Tuzovic M., Reddy S.T., Hendrickson J.E., Lee M.R., Hoffman J., Patel J.K. (2019). Cardiac complications in the adult bone marrow transplant patient. Curr Oncol Rep.

[6] Gaviria J.M., Castro A.F., Restrepo A., Trujillo H., Tapia I., Patiño C. (2000). Nontuberculous mycobacterial infections in hematopoietic stem cell transplant recipients: characteristics of respiratory and catheter-related infections. Biol Blood Marrow Transplant.

[7] Roy V., Weisdorf D. (1997). Mycobacterial infections following bone marrow transplantation: a 20 year retrospective review. Bone Marrow Transplant.

[8] Hoyle C., Goldman J.M. (1994). Life-threatening infections occurring >3 months after BMT. 18 UK bone marrow transplant teams. Bone Marrow Transplant..

[9] Friedrich W., Kern P., Kapp M., Vollebergh P., Haas R.J., Schindewolf C. (1982). T-lymphocyte reconstitution in recipients of bone marrow transplants with and without GVHD: imbalances of T-cell subpopulations having unique regulatory and cognitive functions. Blood.

[10] Mohite U., Patel K., Menon R., Mutha V., Rao P., Ghadage P. (2001). Mycobacterial pulmonary infection post allogeneic bone marrow transplantation. Leuk Lymphoma.

